# Role of HSP60/HSP10 in Lung Cancer: Simple Biomarkers or Leading Actors?

**DOI:** 10.1155/2020/4701868

**Published:** 2020-03-30

**Authors:** Alberto Fucarino, Alessandro Pitruzzella

**Affiliations:** ^1^Department of Biomedicine, Neuroscience and Advanced Diagnostics-University of Palermo, Palermo, Italy; ^2^Consorzio Universitario Caltanissetta, Caltanissetta, Italy

## Abstract

Cancers are one of the major challenges faced by modern medicine both because of their impact in terms of the amount of cases and of the ineffectiveness of therapies used today. A concrete support to the fight against them can be found in the analysis and understanding of the molecular mechanisms involving molecular chaperones. In particular, HSP60 and HSP10 seem to play an important role in carcinogenesis, supporting tumours in their proliferation, survival, and metastasis. Efforts must be directed toward finding ways to eliminate or block this “mistaken” chaperone. Therefore, the scientific community must develop therapeutic strategies that consider HSP60 and HSP10 as the possible target of an anti-tumoural treatment and not only as diagnostic biomarkers, since they contribute to the evolution of pre-cancerous respiratory pathologies in lung tumours. HSP60 acts at the mitochondrial, cytoplasmic, and extracellular levels in the development of cancer pathologies. The molecular mechanisms in which these chaperones are involved concern cell survival, the restoration of a condition of absence of replicative senescence, the promotion of pro-inflammatory environments, and an increase in the ability to form metastases. In this review, we will also present examples of interactions between HSP60 and HSP10 and different molecules and ways to exploit this knowledge in anticancer therapies for lung tumours. In order to improve not only chances for an earlier diagnosis but also treatments for patients suffering from this type of disease, chaperones must be considered as key agents in carcinogenesis and primary targets in therapeutics.

## 1. Introduction

Lung cancer incidence has been increasing in the last years, in both developing and developed countries. It is one of the main causes of death worldwide, and it has become a very frequent malignant tumour for mankind. Although there are several possible ways to treat lung cancer (chemotherapy, radiotherapy, surgery, etc.), the patient survival rate at 5 years is 15% [1]. The survival rate increases when patients are subjected to surgical treatment earlier, but only a small proportion of subjects who have been diagnosed with lung cancer can undergo this procedure [2]. Therefore, it is necessary to optimize diagnostic procedures and to understand the molecular mechanisms of metastasization to reduce the mortality of this pathology. Understanding the molecular mechanism and signalling pathways in lung cancer is also of fundamental importance for the creation of new therapeutic strategies that can assist surgical treatment. Although the number of possible molecular biomarkers is high, the scientific community pays increasing attention to the possible involvement of heat shock proteins in the establishment of lung cancer and its pathological progression. Heat shock proteins (HSPs) are a group of highly conserved proteins that help protect cells from various type of stress (heat, cold, and abnormal levels of glucose or oxygen). They help the correct folding of many proteins and protect cells from deleterious consequences as protein misfolding, premature degradation, or aggregation [3–6]. HSPs normally support other protein functions in normal cells, but they may be present at high levels in cancer cells. This deregulation in the levels of HSPs produced in cancer cells alone could be the cause of metastatic progression, not only in lung tumours but, more generally, in various types of carcinoma [7]. Tumours usually appear as a result of several factors, and, as mentioned above, HSPs should be considered among the genes involved in their progression. This means that some tumours can be considered chaperonopathies. In particular, regarding lung tumours, the scientific evidence of a possible role of HSPs in molecular pathways keeps growing. HSPs localization occurs in various subcellular compartments such as mitochondria, endoplasmic reticulum, microvesicles, and even the nucleus [8]. They can be released out of the cells through different ways (via Golgi or inside extracellular vesicles, such as exosomes), acting as cross cellular messengers. Both a paracrine effect in the proximity of the releasing cell and an endocrine effect through the blood stream are to be considered as possible effector pathways [9]. A massive production of HSPs by neoplastic cells leads this class of proteins to favour the tumour at the expense of the individual [10]. In fact, “pro-tumour” HSPs support cancer cells in different processes, such as their proliferation, growth, and resistance to chemotherapy and radiotherapy treatments, and favour their metastasization [9, 11]. Therefore, the study and development of chaperonotherapy models is of fundamental importance if contextualized within a treatment that already includes classical approaches such as chemo-, radio-, and immunotherapy in order to arrest the progression of tumoural pathology. In addition to the possibility of using HSPs as therapeutic targets in the fight against lung tumours, this protein class appears to be an excellent candidate for predicting disease onset [12–14]. This is of the utmost importance, considering what has been previously stated on the benefits of an early diagnosis of lung tumour.

## 2. HSP 60

The heat shock protein 60 or HSP60 is a protein, weighing of 60 kDa, belonging to the chaperone family. It is used by the cell for the correct folding of other proteins [15]. HSP60 is a highly conserved protein that is present in many species of living organisms [16]. In addition to its main biological function, this class of proteins is the subject of a growing study regarding tumour progression [17, 18]. In different tumour types, the levels of HSPs are altered; therefore, their variation from standard values could be associated to those changes occurring during the processes of carcinogenesis [19].

Under normal conditions, HSP60 and its biological partner (co-chaperonin) HSP10 are two molecular chaperones with mitochondrial localization that, like the other proteins of their family, protect cells from different types of stress, closely related to mitochondrial integrity [20]. HSP60 and HSP10 form a folding cage through their rings and produce large and efficient protein-folding machinery that facilitates proper folding and assembling of mitochondrial imported proteins and corrects misfolded polypeptides [21]. HSP60 assembles into an oligomer with a precise quaternary structure to perform its characteristic role of chaperone. In addition, participation in the process of seven HSP10 subunits and ATP hydrolysis is necessary, with a final assembling of a bell-shaped form [22, 23]. In this review, we are going to illustrate the link between the alterations of HSP60 and HSP10 levels or localization and the occurrence of lung tumours, a relationship which has already been found in other carcinogenic processes such as colorectal, pancreatic, tongue, urinary bladder, prostatic, vesical, and exocervical tumours. It is curious how in some of these tumour forms the levels of HSP60 are increased, while, in others, the expression of the chaperone is reduced [24–44]. This alternation of conflicting results is also maintained when we observe the relationship between HSP60 and the prognosis of several tumours: an apparently anomalous behaviour that can be explained by the dual pro- and anti-apoptotic activity of an over-expression of HSP60. Another property linked to excess of HSP60 is the loss of replicative senescence of tumour cells [45, 46]. Similarly, an increase in synthesis levels of HSP10 is associated with different tumour types. Prostate cancer, exocervical cancer, large bowel cancer, and serous ovarian cancer are all cases in which this condition is present. On the contrary, as with HSP60, it is possible to find examples of tumours in which the levels of HSP10 are reduced, an example being lung cancer [47–51].

Another important factor in the functioning of HSP60 is its location within the cellular compartment [52]. As mentioned, normally, the HSP60/HSP10 complex finds its natural localization within the mitochondrial compartment, while in tumour cells it is not unusual to find HSP60 at the level of the cytoplasm. HSP60 has also been localized in lipid rafts that are rich in cholesterol and glycosphingolipids [26, 53, 54]. Even HSP10 that is normally localized inside the mitochondrial matrix can be found in other localizations, such as microvesicles or cell cytoplasm [55]. It is curious how high extracellular levels of HSP10 can be found during pregnancy. In fact, this is referred to as early pregnancy factor, and there are studies that have shown its importance in cell proliferation and differentiation [56, 57].

As mentioned above, when the chaperonins take the extracellular pathway, they can influence different cytotypes, in particular, HSP60 has an immunomodulatory effect on cells such as macrophages [58, 59] and neutrophils [60, 61]. An increased number of neutrophils and macrophages is a characteristic key to a pathology that usually evolves into lung tumour pathology: the chronic obstructive pulmonary disease (COPD). COPD is a pathology that owes its development mainly to cigarette smoking, and patients affected by this condition are characterized by a progressive and irreversible loss of their lung function. Several epidemiologic studies have showed that, in smokers with COPD, the incidence of lung cancer is five times higher than in smokers without COPD. At the same time, there is a greater ease of onset of lung cancer in patients with a severe level of airway obstruction. The ability of extracellularly released HSPs to modulate the secretions of proinflammatory cytokines probably plays a key role in the progression of COPD itself and in its potential evolution to a carcinogenic level [61–64].

## 3. Lung Cancer: HSP60 and HSP10 Molecular Interactions

Analysing the broad set of proteins with which HSP60 and HSP10 interact, there are several of them that can play a role in the development and progression of lung cancer. However, there are further interactions that may have a protective effect in counteracting the onset of tumours.

### 3.1. Fragile Histidine Triad (FHIT) Protein

**Figure 1:**
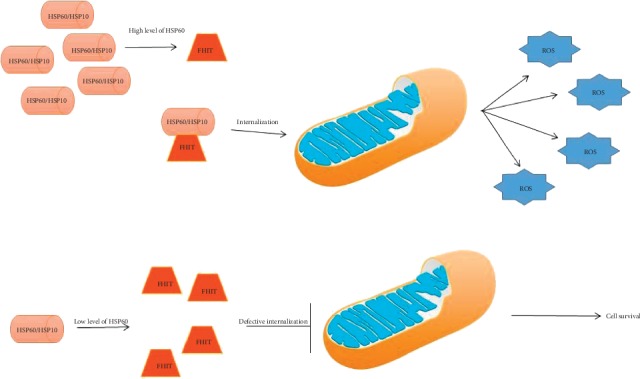
The tumor-suppressor FHIT protein interacts with HSP60, and possibly is folded by the chaperoning complex HSP60/HSP10 inside mitochondria. Therefore, any quantitative or qualitative defect of HSP60 (chaperonopathy by defect) may decrease the level of functional FHIT protein, causing a failure of tumor suppression and favoring carcinogenesis.

Fragile histidine triad protein (FHIT), also known as bis (5′-adenosyl)-triphosphatase, is a member of the histidine triad gene family involved in purine metabolism. FHIT is characterized by an unusual genomic fragility. As a consequence of this condition, its expression is reduced, if not absent, in many cancers. Although the precise function of FHIT is still not fully understood, it is clear that this protein acts as a tumour suppressor. The interaction between FHIT and the molecular chaperone complex HSP60/HSP10 has been demonstrated, and it is probably fundamental for the entry of FHIT into the mitochondria, where it plays an important role in electron transportation. FHIT has been shown to have considerable relevance in lung cancer. Studies conducted in vitro on tumour cells have shown that its expression, associated with a condition of cellular stress, leading tumour cells to trigger apoptotic processes. Furthermore, reduced expression levels, mainly due to genomic damage, have been found in hyperplastic lung lesions, but also in precancerous conditions in those smokers who maintain an apparently normal bronchial epithelium. The entry at the mitochondrial level of FHIT, made possible by the interaction with HSP60/HSP10, is crucial for the antitumour activity of FHIT itself. In fact, through the interaction with Fdxr, at 54-k flavoprotein, it is able to trigger the apoptotic process generating reactive oxygen species (ROS) via p53. A reduced expression of HSP60/HSP10 is therefore linked with a defective mitochondrial internalization of FHIT with consequent loss of apoptotic function by ROS. Otherwise, recent studies have brought to light a new possibility: exploiting the importance of oxidative phosphorylation for a particular cell population, cancer stem cells. Lung tumour cells are sensitive to treatments based on selective inhibitors of oxidative phosphorylation that act on the mitochondrial complex III, while these are ineffective when FHIT is present. Therefore, an assessment of HSP60/HSP10 levels (in association with the expression of FHIT) appears fundamental in choosing the kind of therapy to be used against lung cancer [65–69] (Figure 1).

### 3.2. Toll-Like Receptor (TLR)

As previously stated, the different levels of expression (high or low) of HSP60 present a dichotomy when compared to precancerous conditions, such as COPD, or lung tumours. Human bronchial epithelial cells subjected to a high level of oxidative stress (such as cigarette smoke) increase the release of HSP60 in extracellular compartment. Once HSP60 is released outside the cell, it can bind different receptors present on immune cells, usually promoting an inflammatory state. Among the HSP60 target molecules are toll-like receptors, in particular TLR-4 and TLR-2. Specifically, the expression of TLR-4 is directly associated with the epithelial tissue, allowing it to feed the inflammatory condition via cytokine release. Among the possible ways of activation, the one via MyD88 appears to be the most likely since high levels of this TLR adapter have been found in association with the extracellular release of HSP60. In close association with TLR activation, an increase in extracellular HSP60 levels leads to an upregulation of CREB1 (a nuclear transcription factor) which results in a massive production of IL-8, thus creating the ideal conditions for a proinflammatory environment [62, 70–74]. A condition of this type, often closely associated with continuous external insults such as cigarette smoke, can easily develop a preneoplastic state in lung tumours.

### 3.3. p53/HSP60 Complex

Acting against the inactivation of the replicative senescence is one of the possible strategies to use in the fight against cancerous pathologies. Factors such as oxidative stress, telomere shortening, and DNA damage can trigger a state of replicative senescence that blocks the normal cell cycle. Cancer cells have lost this inhibition to cell proliferation, and the possible restoration of a replicative senescence would guarantee a greater success rate in anticancer treatments. Several studies have demonstrated how important is HSP60 in the loss of replicative senescence in many different tumours. In fact, a reduction of these proteins is associated with the appearance of senescence features and a reduction/arrest of tumour-cell expansion. It is possible to explain this function of chaperonins, if we consider how they are not implicated exclusively in protein folding, but focusing on their antiapoptotic role. Among the proteins with which HSP60 forms complexes, one of the most relevant is certainly p53. The formation of an HSP/p53 complex leads to a reduction in the interaction of p53 itself with the promoters of cell cycle arrest genes, thus preventing the onset of a state of replicative senescence in tumour cells. A further therapeutic strategy could therefore be to modify the interaction domains between HSP60 and p53. Previous studies on mucoepidermoid cell lines in human lungs have already shown the benefits of a doxorubicin treatment that promotes HSP60 acetylation with a consequent reduction of its levels and ability to form a stable complex with p53, leading to a restoration of replicative senescence [29, 46, 75–78].

### 3.4. SAHA (Suberoylanilide Hydroxamic Acid)

The effects of Suberoylanilide hydroxamic acid (SAHA) as an anti-tumour molecule are already known. It is a member of the histone deacetylase inhibitor family (HDACi), and as the name suggests, this class of compounds is involved in the acetylation of histones resulting in a modification of the expression of the chromatin and the consequent transcriptome. SAHA and similar compounds also acetylate other proteins, among these, one of the targets is HSP60. Previous work had already shown that SAHA was able to acetylate other chaperonins such as HSP90 and HSP70. This does not happen, however, for HSP60: in fact, it has been revealed that, in this chaperonin, the induced posttranslational modification is a nitration at the level of the tyrosines 222 and 226, two amino acids present on the apical domain of HSP60. A study conducted on a cell line derived from human lung cancer (H292) has shown that, as a result of this nitration, intracellular HSP60 levels are reduced exclusively at a post-translational level. The nitration of tyrosine probably depends on the reactive nitrogen species created by SAHA activity inside tumoural cells. As a result, there is a reduced capacity of ATP-hydrolysis by HSP60 and an increased difficulty in binding to the co-chaperonin HSP10. In the future, antitumour treatments involving various HDAC is, and SAHA in particular, must take in consideration their ability to interact with HSP60 indirectly, with a pathway that is not linked to their principal mechanism. The property of SAHA to edit post-translational molecules like HSP60 can open new strategies for antitumour therapeutic protocols at a design level [79–84].

### 3.5. Lipid Rafts and Plasma Membrane

In this section, rather than single interactions between HSP60 and target proteins, some anomalous localizations of chaperonin in lung cancer cells will be analysed. On two different cell lines deriving from lung tumours, HSP60 molecules have been detected at the level of the plasma membrane and not at the usual mitochondrial location. The cell lines in question were A549, derived from a human lung adenocarcinoma, and H292, created from a human lung mucoepidermoid. Furthermore, a temporal analysis shows a different presence of HSP60 at the level of the cytoplasmic membrane, probably the result of different stages leading to its secretion. This hypothesis is further confirmed by pointing out that there is an accumulation of HSP60 in the cytoplasmic compartment near the membrane itself, suggesting an active localization of the molecular chaperone in this precise cellular district. Analysing the localization on the plasma membrane of the tumour cells in even more detail, the HSP60s have a preferential aggregation at the level of lipid rafts. Since lipid rafts are often the “departure stations” from which microvesicular bodies originate, the use of treatments with lipid raft pathway inhibitors would reduce the amount of HSP60 released at the extracellular level by cancer cells [55, 85–87].

### 3.6. Pro-Caspase 3

As already explained in the cases analysed above, HSP60 concentration levels can suffer variations, if compared to a basal expression, in case of lung tumours or conditions that can evolve into cancerous diseases (such as COPD). Various examples have been mentioned, both of cases in which expression levels were increased or decreased, and of cases of ectopic localizations. As a result of oxidative stress, tests on mucoepidermoid carcinoma cell lines have shown an increase in the cytoplasmic concentration of HSP60; if paired with a massive release from mitochondrial compartment, the role of HSP60 is pro apoptotic; otherwise, if the mitochondrial release is absent, the chaperone performs an anti-apoptotic action. Studies have shown that HSP60 is able to bind to the inactive form of caspase 3, pro-Caspase 3 (p-C3). This shows how it plays an anti-apoptotic action at the level of lung cancer cells. Indeed, subsequent work has shown that by inhibiting the binding of HSP60 with p-C3, tumour cells prove to be more susceptible to apoptosis. Tumour growth is then interrupted by the activation of the caspase cascade following the activation of p-C3 in caspase 3 (its active form). Therefore, it is necessary to consider the use of drugs and compounds which reduces the binding that stabilizes the inactive form of caspase as a possible therapeutic option, in order to favour a better prognosis and successful treatment in patients suffering from lung cancer [27, 45, 88–90].

## 4. Conclusions

The examples reported in this review and the molecular interactions that will be discovered in the future will constitute an important weapon for doctors, not only in the treatment of lung tumours, as they will also lead to better diagnostic methods, but also the creation of therapies against precancerous conditions that can evolve into lung cancer. Continuous stress conditions in the respiratory mucosa (a clear example is cigarette smoking) increase the levels of chaperonins at the extra-mitochondrial level; the high concentration of HSP60 has a positive effect on cell survival, by inhibiting both apoptotic processes and cellular senescence. This increase in the cytoplasmic levels of the molecular chaperone leads to its release through different mechanisms (multivesicular bodies, exosomes, etc.), feeding a proinflammatory state by acting on the immune cells. Thanks to this positive feedback an ideal environment is created for the development of lung tumours.

The number of cases of individuals with lung cancer is set to increase in the years to come. Both its impact on the world population and its costs on national health systems will be influenced by this trend. The development of new compounds and new therapeutic strategies, associated with an increased knowledge of pathways, will provide a new weapon in the fight against lung tumours, in which the contribution of the HSP60 and HSP10 chaperonins appears increasingly relevant. The time has come to assign a much more important role to these molecular chaperones, making them evolve from simple biomarkers to leading actors in the development and evolution of lung tumours.

## Supplementary Materials

Supplementary Materials Table 1: molecular interactions of HSP60/10 complex in lung cancer cells.
