# Supplementary material for: Role of HSP60/HSP10 in Lung Cancer: Simple Biomarkers or Leading Actors?
Source: J Oncol. 2020 Mar 30;2020:4701868. doi: 10.1155/2020/4701868 (PMC7149434; doi:10.1155/2020/4701868)
Supplement: Supplementary Materials — Table 1: molecular interactions of HSP60/10 complex in lung cancer cells. [file 4701868.f1.docx]

Table 1: Molecular Interactions of HSP60/10 complex in lung cancer cells

| Interaction with | Localisation | Conditions and possible treatments |
| --- | --- | --- |
| FHIT | intramitochondrial | High Level of HSP60: Exploit the internalization in mitochondria of FHIT to increase the production of ROS |
| FHIT | intramitochondrial | Low Level of HSP60: Treatments that inhibit the mitochondrial complex III against cancer stem cells. |
| TLR | cytoplasmic membrane / transmembrane receptors | Extracellularly HSP60: Monitor IL-8 levels and the development of a proinflammatory state |
| p53 | intracellular | Use of post-translational modifications on HSP60 to block p53 binding and restore replicative senescence |
| SAHA | Direct interaction | Nitration at the level of the tyrosines 222 and 226, a reduced capacity of ATP-hydrolysis by HSP60 and an increased difficulty in binding to the co-chaperonin HSP10. |
| p-Caspase3 | Both cytoplasmatic and mitochondrial | High Level of HSP60: cell survival. Limit the binding between HSP60 and pCAS3 to promote apoptosis of tumour cells |

FHIT: Fragile Histidine Triad Protein ; TLR: Toll Like Receptor ; SAHA: Suberoylanilide hydroxamic acid ; ROS: Radical Oxygen Species ; IL-8: Interleukin-8 ; pCAS3: pro-Caspase 3.
